# Short-term outcomes of minimally invasive retromuscular ventral hernia repair using an enhanced view totally extraperitoneal (eTEP) approach: systematic review and meta-analysis

**DOI:** 10.1007/s10029-021-02557-8

**Published:** 2022-01-19

**Authors:** D. Aliseda, C. Sanchez-Justicia, G. Zozaya, J. Lujan, A. Almeida, N. Blanco, P. Martí-Cruchaga, F. Rotellar

**Affiliations:** 1 Department of General Surgery, Clinica Universidad de Navarra, University of Navarra, Av. Pío XII, 36, 31008 Pamplona, Spain; 2 Institute of Health Research of Navarra (IdisNA), Pamplona, Spain

**Keywords:** Enhanced view totally extraperitoneal, eTEP, Minimally invasive surgery, Ventral hernia

## Abstract

**Background:**

The enhanced view totally extraperitoneal (eTEP) approach is becoming increasingly more widely accepted as a promising technique in the treatment of ventral hernia. However, evidence is still lacking regarding the perioperative, postoperative and long-term outcomes of this technique. The aim of this meta-analysis is to summarize the current available evidence regarding the perioperative and short-term outcomes of ventral hernia repair using eTEP.

**Study design:**

A systematic search was performed of PubMed, EMBASE, Cochrane Library and Web of Science electronic databases to identify studies on the laparoscopic or robotic-enhanced view totally extraperitoneal (eTEP) approach for the treatment of ventral hernia. A pooled meta-analysis was performed. The primary end point was focused on short-term outcomes regarding perioperative characteristics and postoperative parameters.

**Results:**

A total of 13 studies were identified involving 918 patients. Minimally invasive eTEP resulted in a rate of surgical site infection of 0% [95% CI 0.0–1.0%], a rate of seroma of 5% [95% CI 2.0–8.0%] and a rate of major complications (Clavien–Dindo III–IV) of 1% [95% CI 0.0–3.0%]. The rate of intraoperative complications was 2% [95% CI 0.0–4.0%] with a conversion rate of 1.0% [95% CI 0.0–3.0%]. Mean hospital length of stay was 1.77 days [95% CI 1.21–2.24]. After a median follow-up of 6.6 months (1–24), the rate of recurrence was 1% [95% CI 0.0–1.0%].

**Conclusion:**

Minimally invasive eTEP is a safe and effective approach for ventral hernia repair, with low reported intraoperative complications and good outcomes.

**Supplementary Information:**

The online version contains supplementary material available at 10.1007/s10029-021-02557-8.

## Introduction

After the first description of laparoscopic ventral hernia repair by Leblanc et al. in 1993, the laparoscopic IPOM (intraperitoneal onlay mesh) technique rapidly became the established approach, as it led to faster recovery and fewer serious wound complications [1]. Subsequently, Agarwal et al. [2] improved the IPOM technique introducing defect closure. The defect closure performed in IPOM + reduced seroma rates [3] recurrence and bulging [4]. However, complications involving intraperitoneal mesh placement such as mesh adhesions, fistulation and migration became a problem. In addition, increased postoperative pain, in relation to mesh fixation [5], and higher reoperation rates [6] led to a significant drop in the number of laparoscopic IPOM repairs (from 33.8% in 2013 to 21.0% in 2019) [7]. As a result, there has recently been an increase in new minimally invasive approaches such as eTEP [7].

Described by Daes in 2012 for laparoscopic inguinal hernia, it was later adapted for ventral hernia repair, resulting in a technically challenging and demanding approach [8]. This technique positions the mesh in the retro-rectus space, without entering the abdominal cavity, to overcome the complications aforementioned and improve outcomes [7, 9]. Belyansky et al. published the first outcomes in 2018 from a multicenter study that showed the feasibility and safety of this ventral hernia repair technique [10].

Since then, various articles have been published reporting the outcomes of this approach for ventral hernia repair [11]. Despite this, studies with solid evidence supporting this technique are lacking. The objective of this study is to perform a systematic review of the literature and meta-analysis to summarize and ascertain the safety and short-term outcomes of eTEP for ventral hernia repair.

## Material and methods

This systematic review was conducted in accordance with the latest updated Preferred Reporting Items for Systematic Reviews and Meta-Analyses (PRISMA) guidelines [12]. This review and the protocol were registered in the PROSPERO platform (CRD42021231029).

### Search strategy

The PubMed/MEDLINE, EMBASE, Cochrane Library and Web of Science electronic databases were reviewed using the following search strategy combining keywords and using Boolean operators. The following key terms were used to identify relevant studies: “Rives Stoppa”, “retro rectus” “retromuscular”, “sublay repair”, “enhanced view total extraperitoneal”, “laparoscopic”, “minimally invasive”, “robotic”, “hybrid”, “ventral hernia” and “incisional hernia”. All possible combinations of keywords were utilized. An additional search was conducted using bibliographic cross-referencing. The final search was performed in January 2021.

### Study selection

The inclusion criteria were studies written in English or Spanish published between January 2005 and January 2021 that included adults over the age of 18 years diagnosed with ventral hernia (primary or incisional) according to the European Hernia Society (EHS) classification [13]. Patients undergoing concomitant procedures, or inguinal or parastomal hernia repair were excluded. The hernia repair had to be performed using a laparoscopic or robotic approach and using the enhanced view totally extraperitoneal technique as proposed by Belyanski et al. [10]. All the studies had to include a good description of technique and end points. Reviews, editorials, and case reports of < 5 patients were excluded, as were manuscripts in which other minimally invasive hernia repair techniques were performed. When duplicates were detected, the largest series was selected.

All the articles retrieved were screened in duplicate. Two researchers (D.A and C.S-J) carried out the first blind screening in duplicate by reading titles and abstracts. The subsequent identification of articles to be included was performed by reading full texts, also in duplicate (D.A and C.S-J). In the process of identifying articles to be included, the rejected articles were correctly identified and the lack of fulfillment of the inclusion criteria was appropriately indicated (Supplementary material Table 1S). In the event of inclusion/exclusion discrepancies (both in screening and identification), a third investigator (P.M-C) was appointed as a referee, or any doubts were resolved by group consensus.

### Data extraction and quality assessment

Data extraction was blinded and performed in duplicate (D.A and A.A) using a data extraction form specifically developed for the review, which was then cross-checked. From each study, at least the following items were extracted: title of the review, first author, year of publication, the authors’ affiliation country, patient baseline characteristics, perioperative details, postoperative outcomes and follow-up. The methodological quality of the selected studies was assessed by D.A and an experienced external epidemiologist using Methodological Index for Non-randomized Studies (MINORS) criteria.

### Statistical analysis

This meta-analysis was conducted with STATA version 16 (StataCorp, College Station, Texas 77845 USA). Continuous data were expressed as means or medians with standard derivations or ranges, as appropriate. Also, categorical variables were shown as numbers and percentages for descriptive purposes. The “*metan*” and “*metaprop*” programs were used to pool means and proportions with 95% confidence intervals, respectively. The STATA “*metaprop*” program allows the execution of meta-analyses of binomial data using the score statistic and the exact binomial method and includes the Freeman–Tukey double arcsine transformation of proportions by stabilizing between-study variance [13]. It is also a convenient method for dealing with proportions near the boundary (0 or 1). Otherwise, studies would be excluded from the analysis resulting in a biased pooled estimate [13]. Furthermore, pooled proportions were derived from random effect models (because we assumed that the true effect estimated in each study varied due to differences in patient characteristics and assay types). Heterogeneity across studies was assessed with Cochran’s *Q* test, and based on the method reported by DerSimonian and Laird substantial significance was set when the *p* value was < 0.10. An *I*-square value of < 25% was defined to represent low heterogeneity, a value between 25 and 50% was defined as moderate heterogeneity, and a value > 50% was defined as high heterogeneity.

A univariate meta-regression was also conducted with the “*metareg*” function. Analysis was done in a random effect-restricted maximum likelihood model with the Knapp–Hartung variance estimation accompanied by the use of a *t* distribution in place of a normal distribution [14]. Univariate regression was considered for perioperative and short-term outcomes (dependent variable) with high heterogeneity (*I*^2^ > 50%) and corrected for conventional factors that could result in the variability in these outcomes (independent variables). Publication bias was visually explored using funnel plots, quantitively assessed using Egger’s test and was considered to exist when *p* < 0.10. In the case of articles only reporting the median, range and the size of the sample, to pool data, mean values and standard deviations were calculated using the formulas proposed by Hozo SP et al. [15]. All tests were two-sided with a significance level of 0.05.

### Measures and endpoints

Primary end points were short-term outcomes, in terms of:

#### Perioperative characteristics

These included intraoperative complications (using the Delphi study definition [16] and including conversion) conversion rate, length of hospital stay and operative time.

#### Postoperative parameters

These included wound complications (surgical site infection, seroma and hematoma), postoperative major morbidity (Clavien–Dindo III–IV [17]), reoperation, readmission and recurrence rate.

## Results

### Search results

**Fig. 1:**
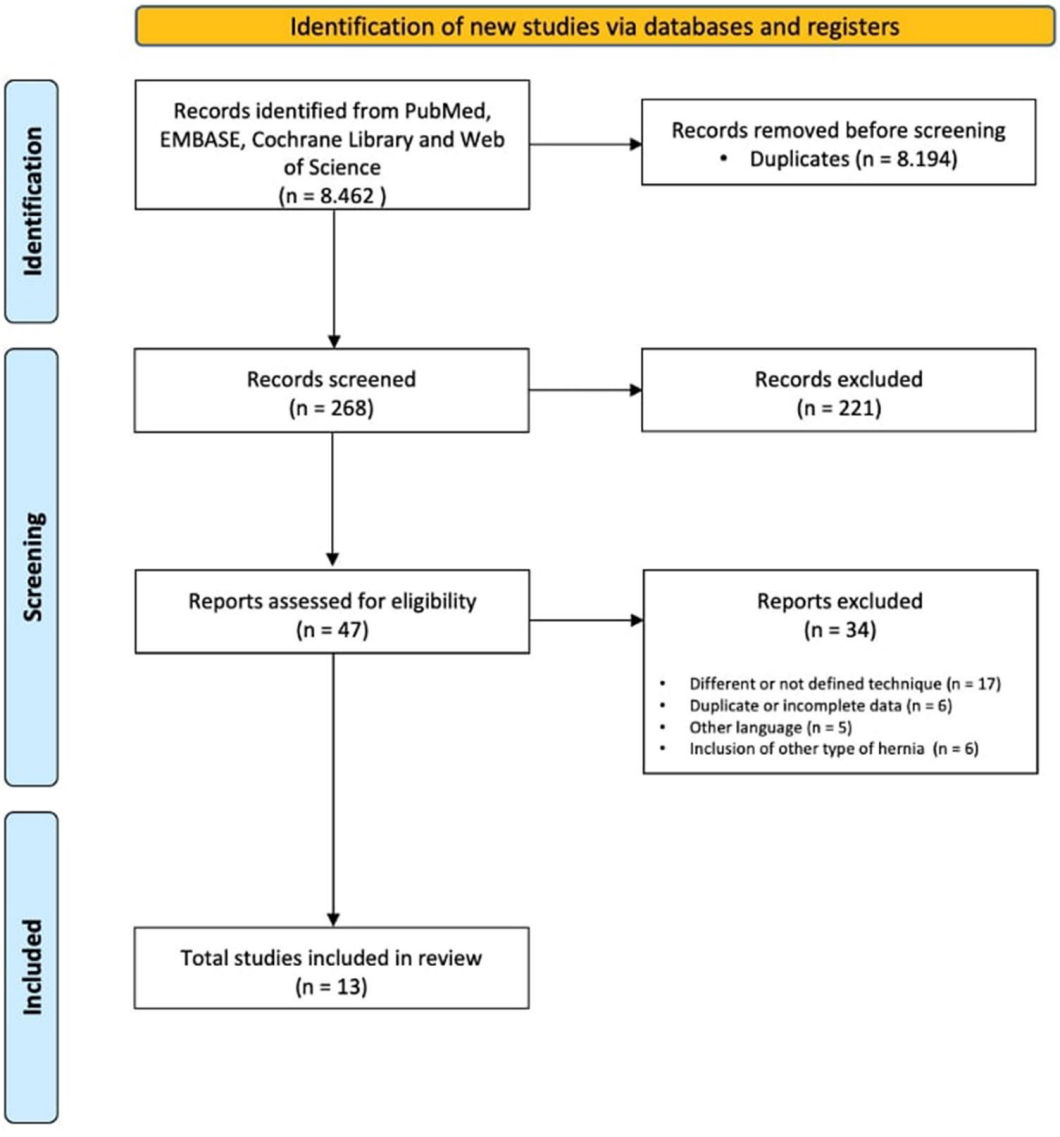
Flowchart of study screening according to PRISMA guidelines

A total of 8,462 studies were retrieved by the literature search. After removing duplicates, 268 articles were screened. No other studies were found via citation searching. A full-text review was performed on 47 reports from the electronic searches. Finally, 13 reports met the inclusion criteria for this systematic review (Table 1). The 2020 PRISMA flowchart with each step of the selection process is presented in Fig. 1.

**Table 1:** Selected studies reporting the minimally invasive eTEP approach for ventral hernia repair

Author	Year	Country	No. of patients	Study design	Center	MINORS score
Kumar N et al.[11]	2020	India	46	Prospective	Single	19/24
Ngo P et al.[27]	2020	France	112	Prospective	Multi	8/16
Salido S et al.[18]	2020	Spain	40	Prospective	Multi	11/16
Prakhar G et al.[22]	2020	India	171	Retrospective	Single	7/16
Mitura K et al. [19]	2020	Poland	11	Prospective	Single	9/16
Sanna A et al. [23]	2020	Italy	18	Retrospective	Single	9/16
Morrell ALG et al.[24]	2020	Brazil	74	Retrospective	Multi	11/16
Kudsi OY et al.[20]	2020	USA	82	Retrospective	Single	17/24
Köhler G et al.[25]	2019	Austria	31	Retrospective	Single	11/16
Baig SJ et al.[28]	2019	India	21	Retrospective	Single	7/16
Penchev D et al.[26]	2019	Bulgaria	27	Retrospective	Single	17/24
Lu R et al.[21]	2019	USA	206	Retrospective	Single	18/24
Belyansky I et al. [10]	2017	USA	79	Retrospective	Multi	10/16

*eTEP* enhanced view totally extraperitoneal

### Study characteristics

Within the 13 studies included, there were 12 observational studies (including one propensity-score study) and 1 interventional study [11]. Four studies were prospective (3 observational; 1 interventional) and nine were retrospective series (Table 1). Preoperative, perioperative and short term-postoperative characteristics of patients included in the studies are provided in Supplementary material Tables 2S, 3S and 4S.

A total of 918 patients from 12 institutions were included in this study. The study population was composed of 467 men and 451 women. Overall, patient mean age was 54.29 years (± 1.28) with a mean BMI of 29.16 kg/m^2^ (± 0.58) with five studies [10, 18–21] presenting an obese population with a mean BMI ≥ 30 kg/m^2^ (Supplementary material Table 2S). Nine studies [11, 18–20, 22–26] reported the hernia etiology, with 45.7% primary ventral hernia. Nine studies [11, 18, 19, 22, 23, 25–28] provided data on hernia location according to the EHS classification [13] resulting in 96.2% of midline hernias and 3.8% of lateral hernias. Hernia size (width) was reported in eight studies [11, 18–21, 24, 25, 28] and resulted in a mean size of 6.38 cm [5.12–7.63]. Information regarding covered mesh area is shown in Supplementary material Table 2S.

### Perioperative characteristics

**Fig. 2:**
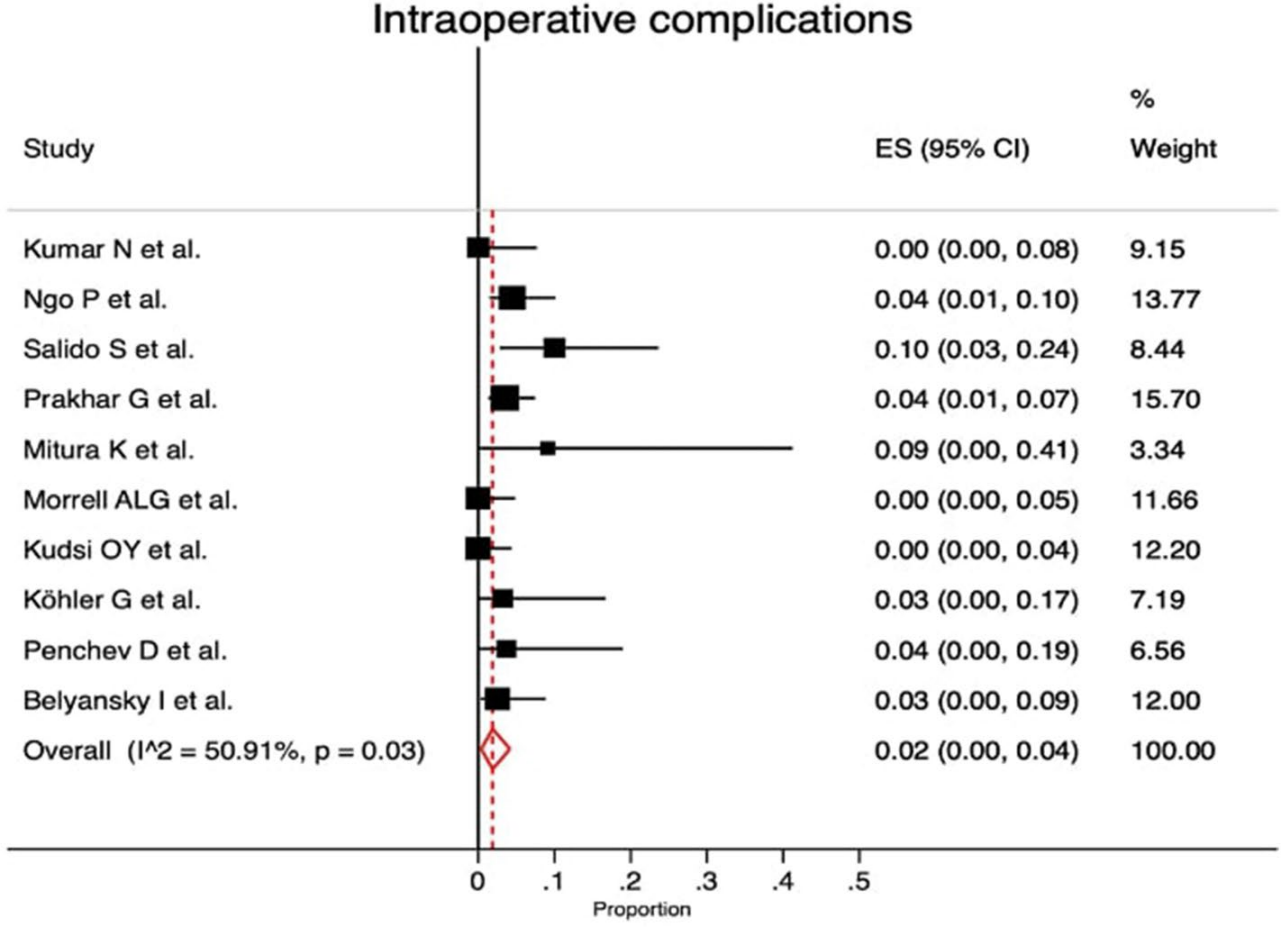
Forest plot of intraoperative complication rate

**Fig. 3:**
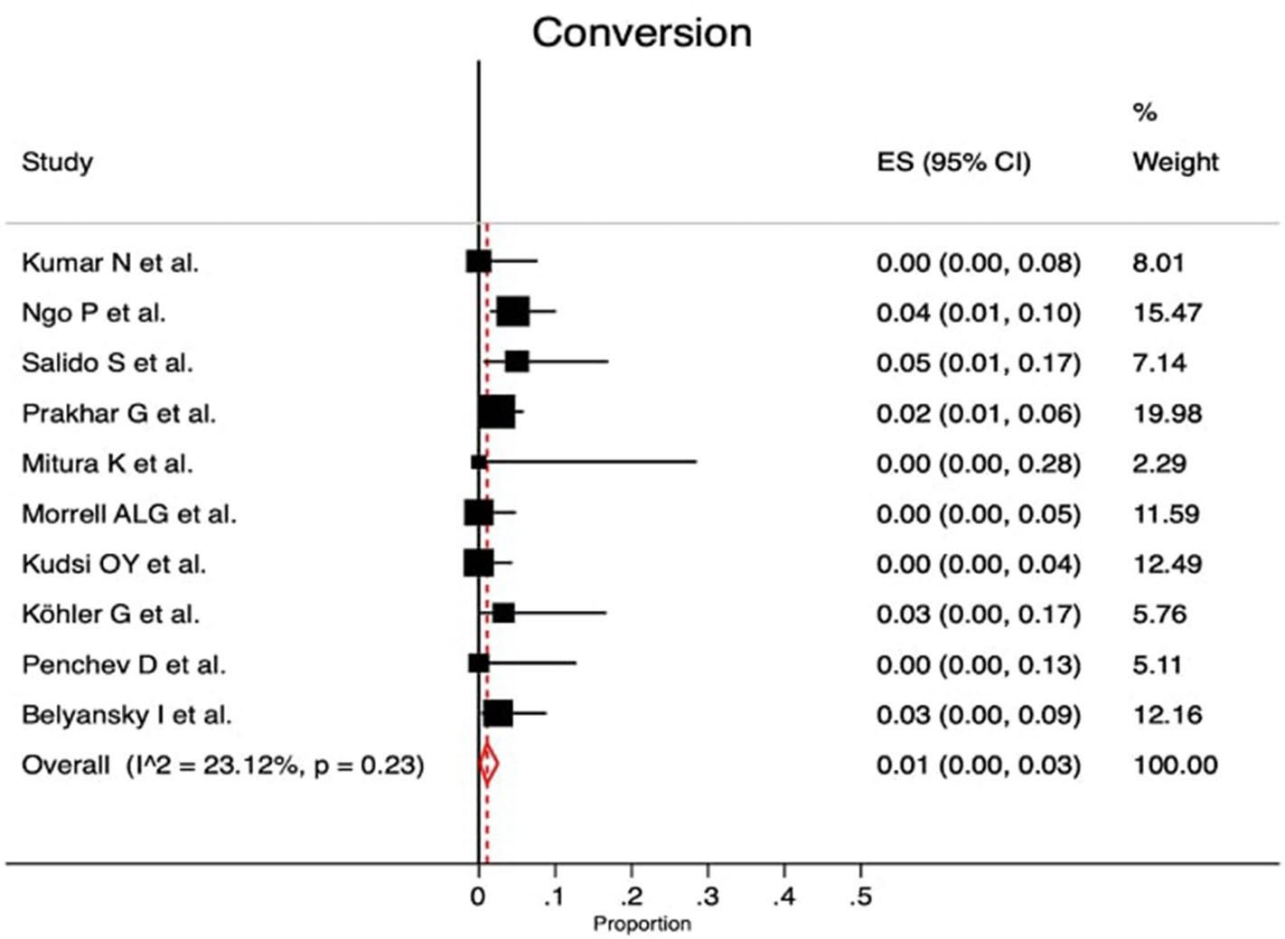
Forest plot of conversion rate

Laparoscopic eTEP was performed in ten studies, two studies reported totally robotic approach and one study showed the results of robotic and laparoscopic eTEP (Supplementary material Table 2S). The proportion of intraoperative complications was given in 10/13 studies (heterogeneity *p* value = 0.03; *I*^2^ = 50.91%; with a proportion of 2% [95% CI 0.0–4.0%]) (Fig. 2). The conversion rate was reported in 10/13 studies (heterogeneity *p* value = 0.23; *I*^2^ = 23.12%; with a proportion of 1.0% [95% CI 0.0–3.0%]) (Fig. 3). Supplementary Table 2S shows that the mean operative time was 148.89 min [95% CI 129.45–168.34] and length of hospital stay was 1.77 days [95% CI 1.21–2.24]. Within this review, in 117 cases (12.7%) (Supplementary material Table 3S) transversus abdominis release (TAR) was performed in association with RS mainly due to large defects or excessive tension on the posterior layer.

### Postoperative characteristics

**Fig. 4:**
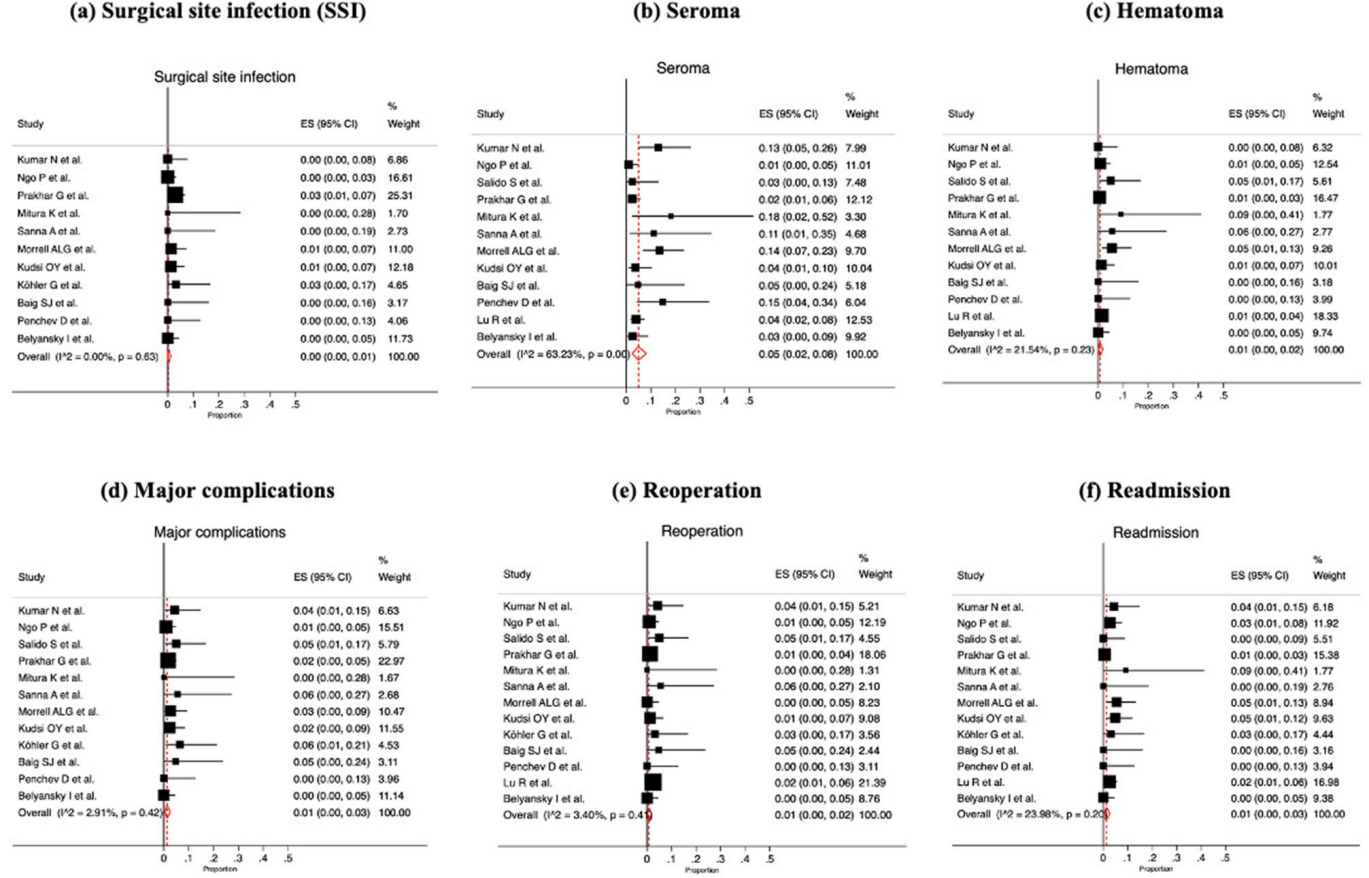
Meta-analysis of short-term outcomes

The rate of surgical site infection was reported in 11/13 studies (heterogeneity *p* value = 0.63; *I*^2^ = 0.0%; with a rate of 0% [95% CI 0.0–1.0%]) (Fig. 4a). In terms of surgical site occurrence (SSO), seroma and hematoma were evaluated. Twelve reports assessed seroma rates (heterogeneity *p* value = 0.000; *I*^2^ = 63.23%; with a rate of 5% [95% CI 2.0–8.0%]) (Fig. 4b) and 12/13 studies reported hematoma rates (heterogeneity *p* value = 0.23; *I*^2^ = 21.54%; with a rate of 1% [95% CI 0.0–2.0%]) (Fig. 4c). The rate of major complications (Clavien–Dindo III–IV) was calculated in 12/13 studies (heterogeneity *p* value = 0.42; *I*^2^ = 2.91%; with a rate of 1% [95% CI 0.0–3.0%]) (Fig. 4d). The reoperation rate was reported in 13/13 (heterogeneity *p* value = 0.41; *I*^2^ = 3.40%; with a rate of 1% [95% CI 0.0–2.0%]) (Fig. 4e). The readmission rate was provided in 13/13 (heterogeneity *p* value = 0.20; *I*^2^ = 23.98%; with a rate of 1% [95% CI 0.0–3.0%]) (Fig. 4f).

**Fig. 5:**
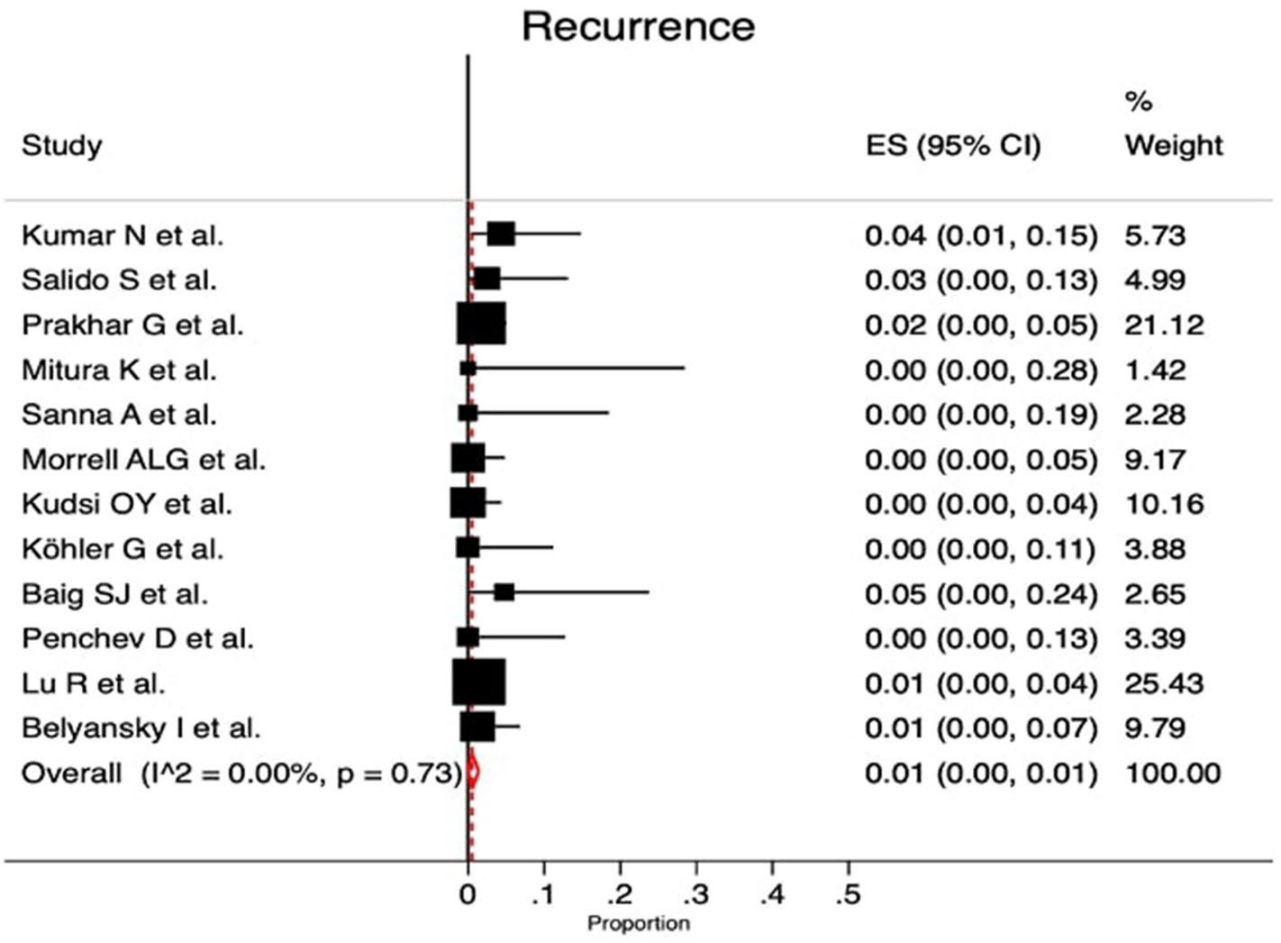
Forest plot of recurrence rate

Follow-up ranged from 1 to 24 months with a median of 6.6 months (Supplementary material Table 4S). Twelve studies reported recurrence (heterogeneity *p* value = 0.73; *I*^2^ = 0.0%; with a rate of 1% [95% CI 0.0–1.0%]) (Fig. 5).

### Meta-regression analysis

A meta-regression was performed to explore sources of heterogeneity in intraoperative complication and seroma rates including: minimally invasive (MI) approach (laparoscopic vs robotic), study size (< 50 vs. ≥ 50 patients), age (mean age < 55 vs. ≥ 55 years) and obesity (mean BMI < 30 vs. ≥ 30 kg/m^2^). Meta-regression analyses determined that MI approach did not show a statistical trend for intraoperative complications rate (β =  − 0.0387; SE: 0.0910; *p* = 0.682) and seroma rate (β = 0.0407; SE = 0.0879; *p* = 0.653). Moreover, study size did not show a statistical trend for intraoperative complication rate (β =  − 0.0387; SE: 0.0910; *p* = 0.682) and seroma rate (β = 0.0407; SE = 0.0879; *p* = 0.653). Neither age (β = − 0.0147; SE = 0.0720; *p* = 0.842) nor BMI (kg/m^2^) (β =  − 0.0214; SE = 0.0670; *p* = 0.755) was significantly identified as confounding factors in seroma rates. The same results were found in terms of intraoperative complications rates where age (β = 0.0140; SE = 0.0774; *p* = 0.861) and BMI (kg/m^2^) (β = 0.0050; SE = 0.0826; *p* = 0.953) were not significantly associated as confounding factors.

### Publication bias analysis

All the studies were independently assessed for bias in each variable using funnel plots and Egger’s test (Supplementary material Figure S1–S9B). Publication bias was found for seroma rates (Supplementary material Figure S4B).

### Methodological quality of studies

The comparative and the non-comparative studies achieved a median MINORS score of 17.5/24 (range 17–19) and 9/16 (range 7–11), respectively. The MINORS score evaluation for each study is shown in Supplementary material (Table 5S).

## Discussion

This is, to our knowledge, the first systematic review and meta-analysis that provides a comprehensive overview of the available evidence regarding the feasibility, safety and short-term outcomes of eTEP for ventral hernia repair.

### Perioperative characteristics

The eTEP is a set of maneuvers and operative strategies which was developed to overcome the limits of the TEP approach for inguinal hernia and was later adapted for the surgical treatment of medial and lateral ventral hernias [8]. These maneuvers are able to broaden the extraperitoneal space in a minimally invasive approach. The eTEP is a challenging technique that requires a thorough knowledge of the anatomy of the extraperitoneal space and where advanced laparoscopic skills are needed to perform some maneuvers successfully [29]. In addition, there are multiple approaches and technical aspects to consider when approaching ventral hernia repair with eTEP [29].

As a consequence of the difficulty of this technique as well as its recent implementation, several articles reported a longer operative time for the eTEP approach compared with other minimally invasive ventral hernia repair techniques [30]. However, once the learning curve is completed, a decrease in operative times and adverse outcomes is observed [31].

Despite the complexity of this technique, according to this study eTEP seems to be safe in terms of intraoperative events. The rate of intraoperative complications and conversion in this meta-analysis was 2.0% and 1.0%, respectively. These results appear to be similar compared with laparoscopic IPOM and seems to improve those derived from sublay OVHR [32]. Not entering the abdominal cavity is a favorable characteristic of this technique, avoiding adhesiolyisis and its associated complications [33, 34]. In fact, eTEP seems to improve one of the problems associated with IPOM and IPOM + , avoiding mesh fixation with tacks or transabdominal sutures and thus decreasing postoperative pain [5]. However, a note of caution must be sounded, since the risk of bowel injury is still present due to thermal injury behind the posterior layer and also during the crossover maneuver, especially in patients who have had previous abdominal surgery [18].

Recent studies have shown a reduction of pain and a better functional recovery when comparing eTEP with IPOM + [11, 35]. This pain reduction due to unnecessary mesh fixation may be one of the reasons for the differences in length of hospital stay between IPOM and eTEP. In this study, hospital stay was 1.77 days, which appears to be in line with other transabdominal extra- and intraperitoneal minimally invasive ventral hernia repair techniques [20, 35] and seems to improve over OVHR [36], including open sublay technique[32].

In addition to these advantages, the vast majority of studies reporting on eTEP approach used uncoated polypropylene meshes. These meshes are affordable, thus decreasing the costs of the procedure compared to the IPOM, where much more expensive meshes with an adhesion barrier are commonly used [37].

Another interesting aspect to consider is the irruption of robotic surgery for eTEP. The introduction of robotic approach has allowed to optimize some maneuvers and subsequently operate more complex patients [21]. The three-dimensional view and freedom of movement that the robotic approach introduces may help in those technically difficult maneuvers and in intracorporeal suturing. These benefits lead to shorter lengths of stay with similar morbidity as compared to laparoscopic approaches as Warren JA et al. have reported [38].

### Postoperative characteristics

During the dissection of the retromuscular plane, a large space is created with the consequent risk of seroma and hematoma formation. In the literature, the rates of seroma and hematoma formation of LVHR differ significantly between studies with studies reporting a significantly higher seroma rate of IPOM + compared with eTEP and vice versa [11, 35]. Anyway, more studies are needed to clarify this matter.

There are several specific complications related to the eTEP approach. Such complications as injury to the linea alba, retromuscular hematoma or injury to the neurovascular bundle could—theoretically— increase morbidity and reoperation rates especially at the beginning of the learning curve [10]. Recently, Henriksen et al. [6] showed a rate of reoperation of 5.0% and 2.7% of OVHR and laparoscopic IPOM, respectively. Furthermore, a large propensity score-matched comparison of almost 10,000 patients showed postoperative surgical complication rates of a 3.4% and 10.5% after laparoscopic IPOM and sublay OVHR, respectively [32]. Therefore, based on this meta-analysis, in the hands of well-trained hernia surgeons, the eTEP approach seems to be safe in terms of major complications and reoperation. However, we firmly believe that it is of utmost importance to emphasize the need for adequate preparation before engaging in any of these complex repairs. As the pioneers of the eTEP approach point out, training is of vital importance to perform safe surgery with good results [29].

A recent meta-analysis of 51 articles showed that retromuscular mesh repair is associated with a lower recurrence rate [39]. However, dissecting the retromuscular plane requires the correct restoration of this space prior to the placement of the mesh. During eTEP, the incomplete closure of the posterior layer due to technical demanding maneuvers or the breakdown of the posterior layer because of increased tension are specific limitations that may increase recurrence and complications [11]. For this reason, after appropriate training, it is recommended to start using this technique by repairing small midline hernias associated with diastasis recti [29]

The rate of recurrence after one-year follow-up of laparoscopic IPOM and sublay OVHR according to Kökerling et al. [32] was 4.2% and 4.1%, respectively. Based on this meta-analysis, eTEP seems to be effective in the repair of ventral hernia with a low recurrence rate. However, the follow-up period of this study is short, so that to correctly assess recurrence, studies with a larger follow-up would be needed to confirm this result.

### Limitations

The limitations of this systematic review and meta-analysis are related to the small number of published studies on this technique as well as the heterogeneity of the studies, the sample size and the observational design of most studies included. Another aspect to bear in mind is the short follow-up to evaluate recurrence and the difference in surgical experience, between studies, with the MI and eTEP technique. Furthermore, it should be taken into account that the surgeries included in this study were performed by expert surgeons in the field of minimally invasive abdominal wall surgery and some of them are even pioneers in the eTEP approach for ventral hernia. For this reason, the good results of this study should be considered in a balanced way and these aspects should be taken into account when assessing its reproducibility. Despite its limitations, the present study offers an objective summary of the evidence of MI eTEP approach for ventral hernia repair that could be useful in guiding surgical decisions.

## Conclusion

This systematic review and meta-analysis summarize the perioperative and postoperative short-term outcomes of minimally invasive eTEP. The outcomes observed in this meta-analysis suggest that, in the hands of well-trained hernia surgeons, minimally invasive eTEP is a safe and effective approach for ventral hernia repair. Despite this, large randomized clinical trials are needed to evaluate the short- and long-term outcomes of this new technique.

## Supplementary Information

Below is the link to the electronic supplementary material.

Supplementary file1 (DOCX 2377 KB)
