# Supplementary material for: Short-term outcomes of minimally invasive retromuscular ventral hernia repair using an enhanced view totally extraperitoneal (eTEP) approach: systematic review and meta-analysis
Source: Hernia. 2022 Jan 19;26(6):1511–20. doi: 10.1007/s10029-021-02557-8 (PMC9684241; doi:10.1007/s10029-021-02557-8)

**SUPPLEMENTARY MATERIAL**

1. PUBLICATION BIAS TEST FOR THE META-ANALYSIS

**INTRAOPERATIVE COMPLICATIONS**

(Supplementary material. 1)

S1A. Data of Egger’s test


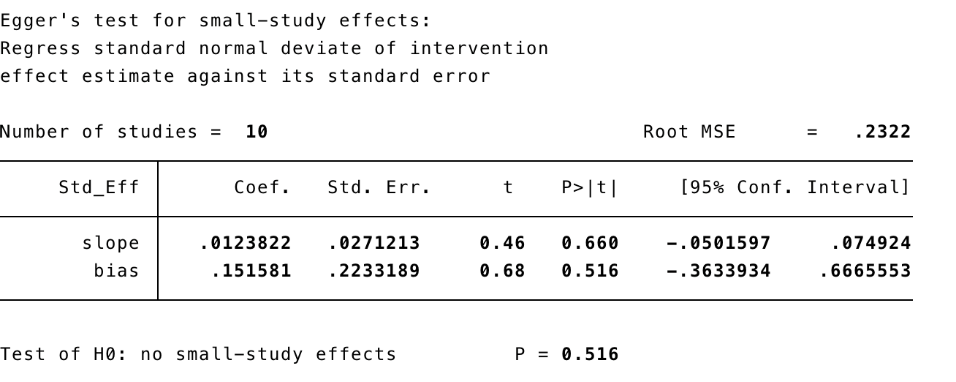


S1B. Funnel plot showing publication bias; intraoperative complications % (X-axis) with it is standard error (Y-axis)

**
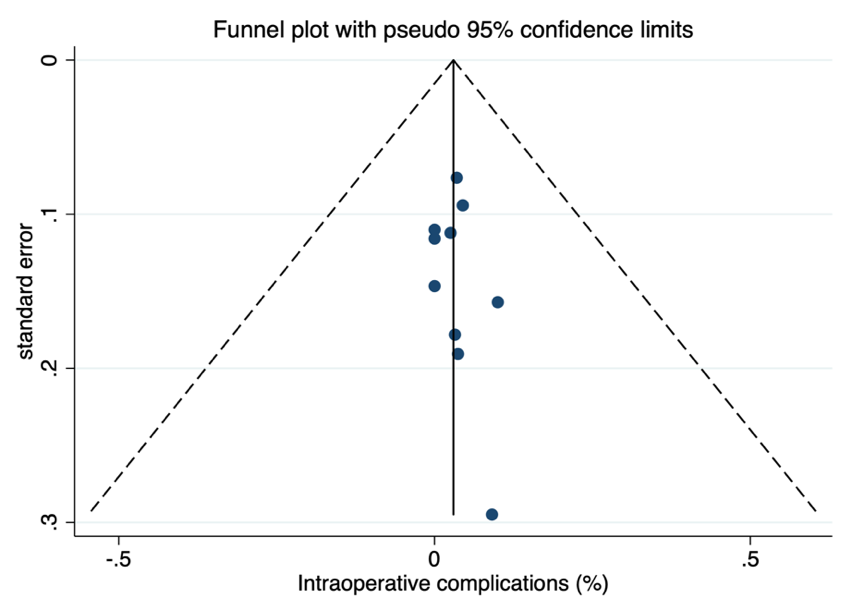
**

**CONVERSION RATE**

(Supplementary material. 2)

S2A. Data of Egger’s test


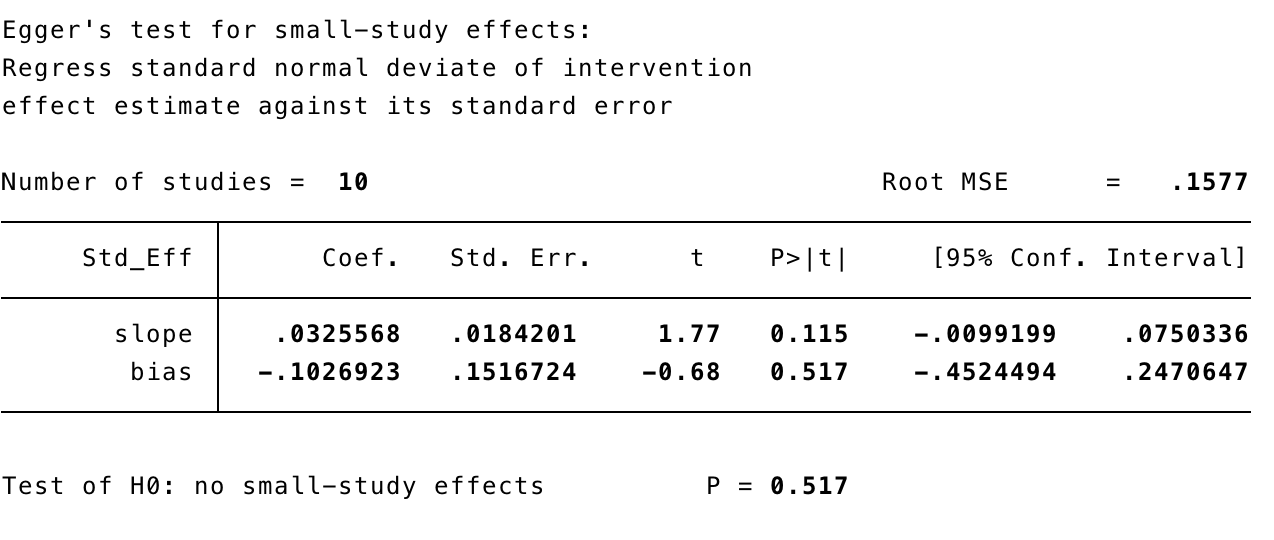


S2B. Funnel plot showing publication bias; conversion rate % (X-axis) with it is standard error (Y-axis)


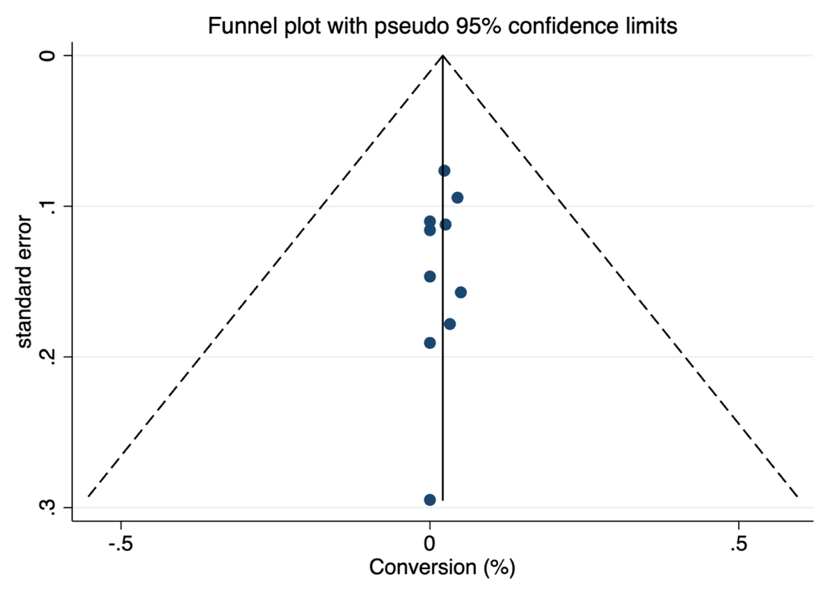


**SURGICAL SITE INFECTION RATE**

(Supplementary material. 3)

S3A. Data of Egger’s test


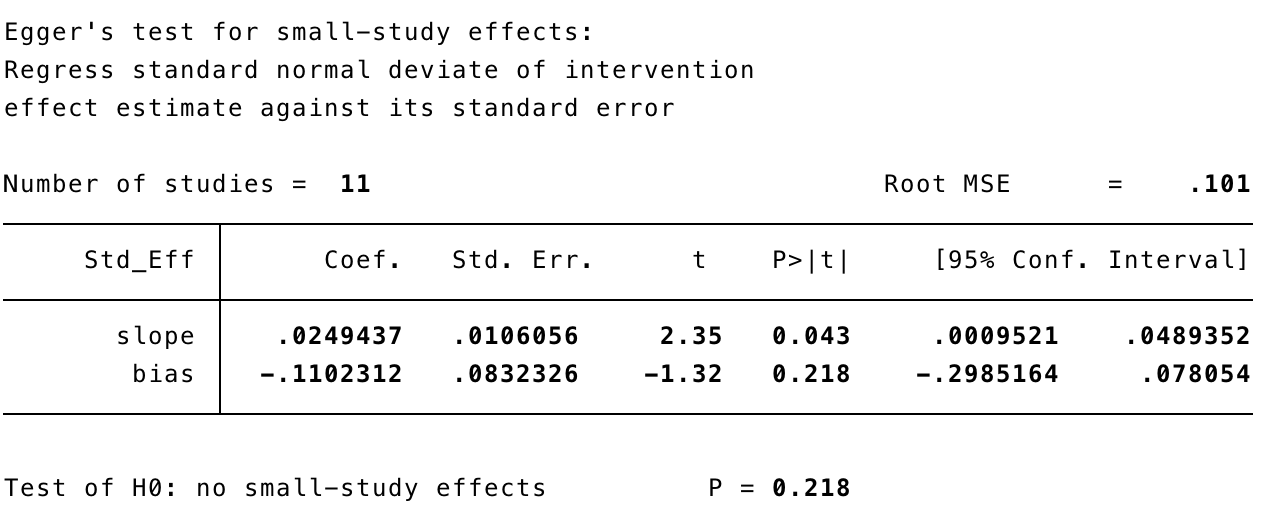


S3B. Funnel plot showing publication bias; surgical site infection % (X-axis) with it is standard error (Y-axis)


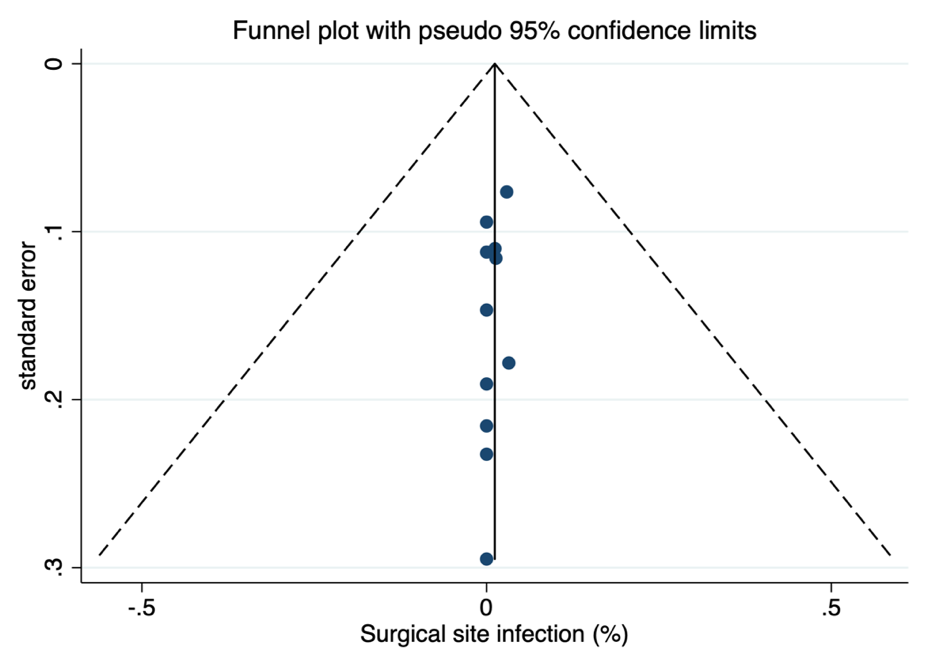


**SEROMA RATE**

(Supplementary material. 4)

S4A. Data of Egger’s test

**
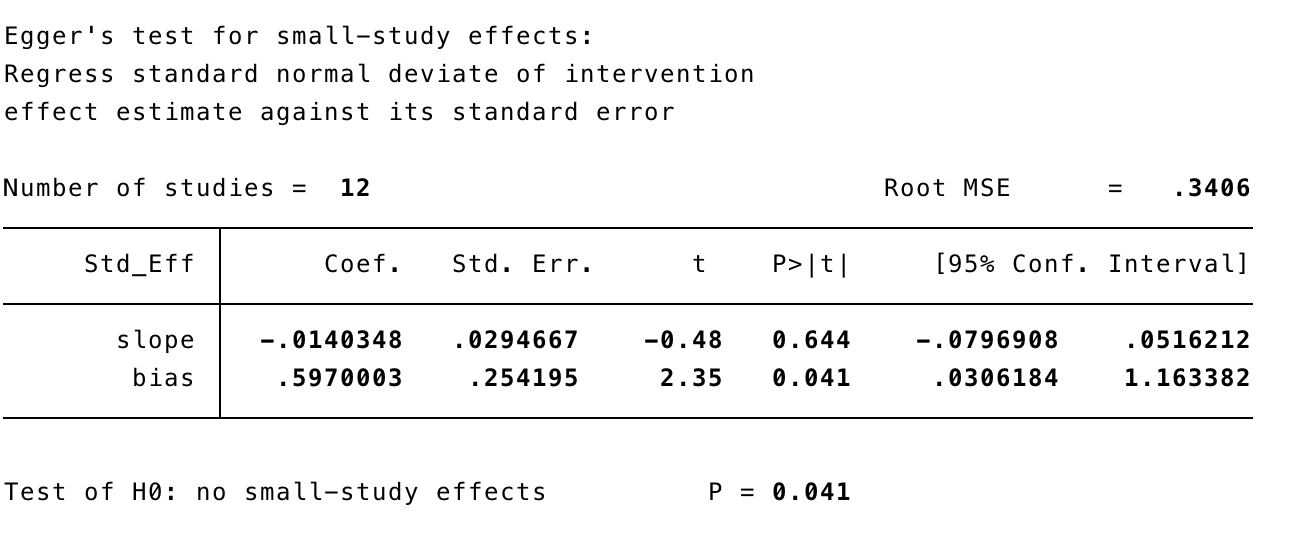
**

S4B. Funnel plot showing publication bias; seroma % (X-axis) with it is standard error (Y-axis)

**
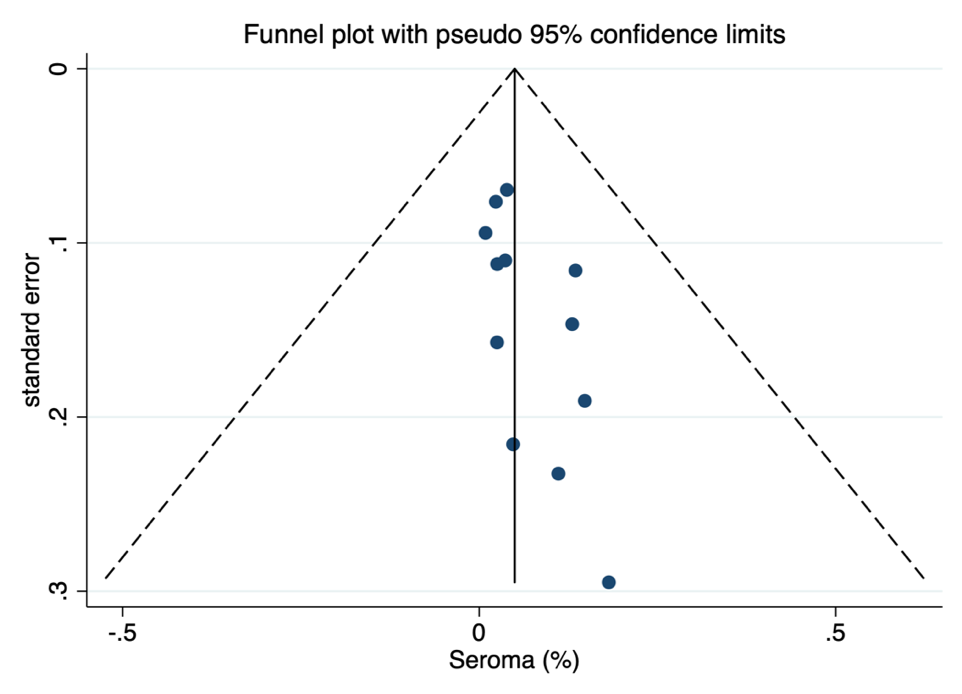
**

**HEMATOMA RATE**

(Supplementary material. 5)

S5A. Data of Egger’s test


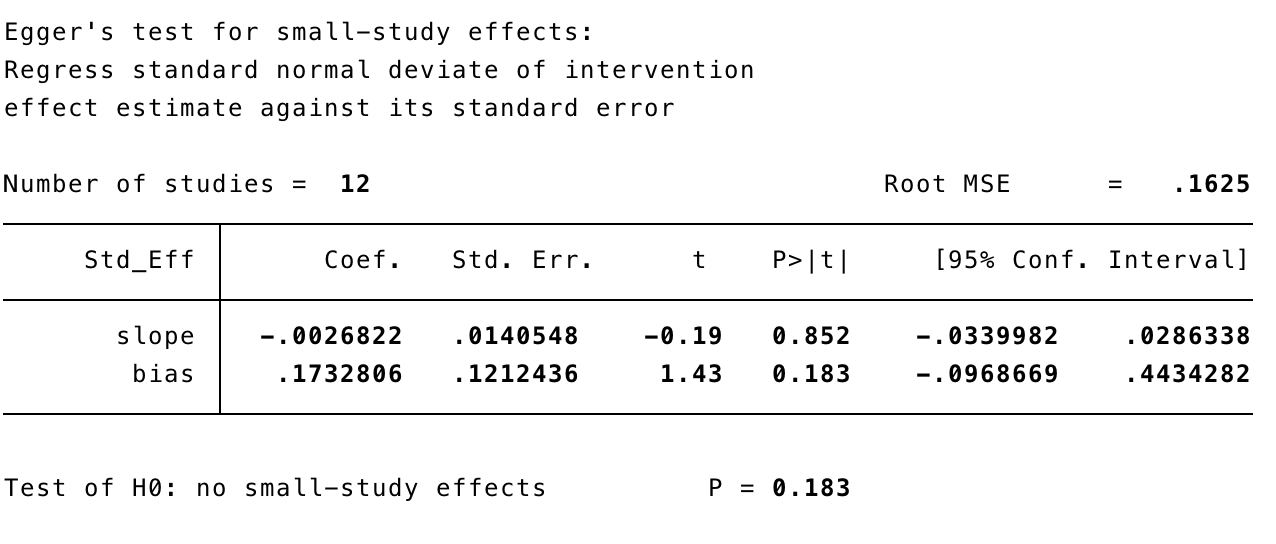


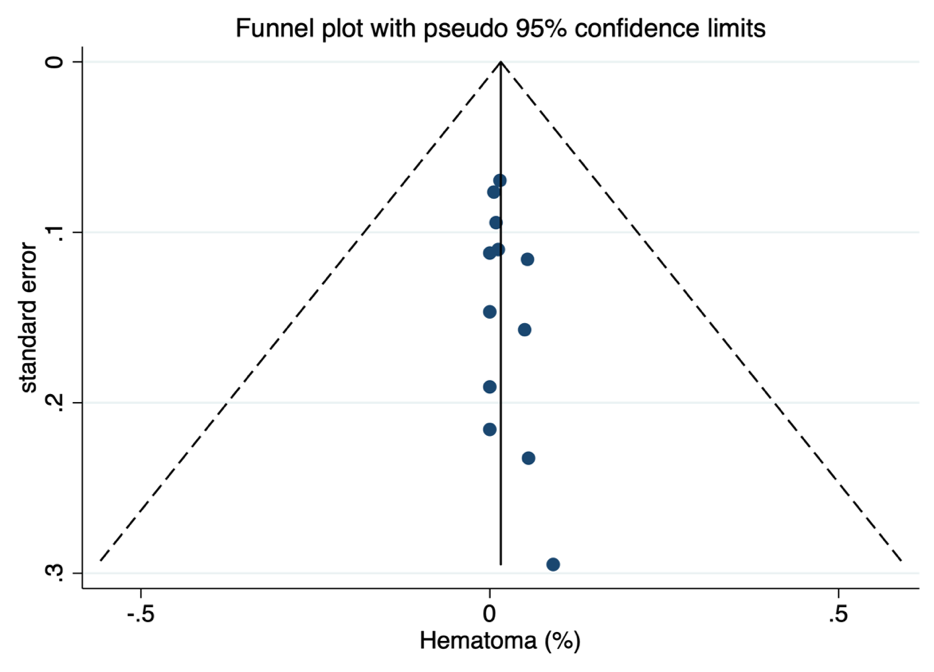
S5B. Funnel plot showing publication bias; hematoma % (X-axis) with it is standard error (Y-axis)

**MAJOR COMPLICATIONS**

(Supplementary material. 6)

S6A. Data of Egger’s test


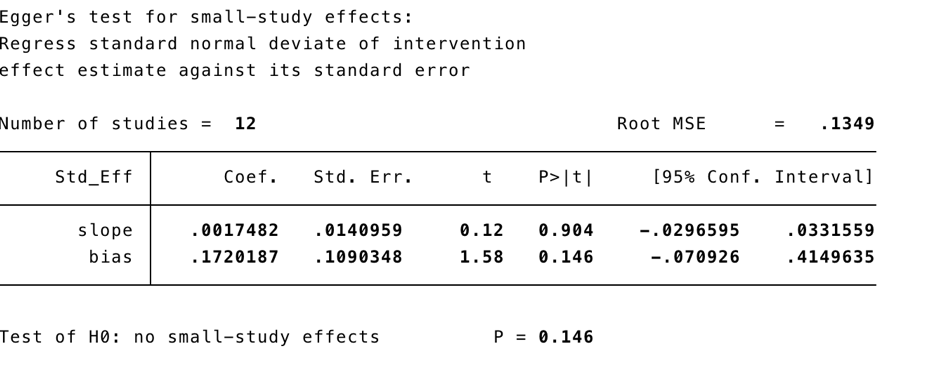


S6B. Funnel plot showing publication bias; major complications % (X-axis) with it is standard error (Y-axis)


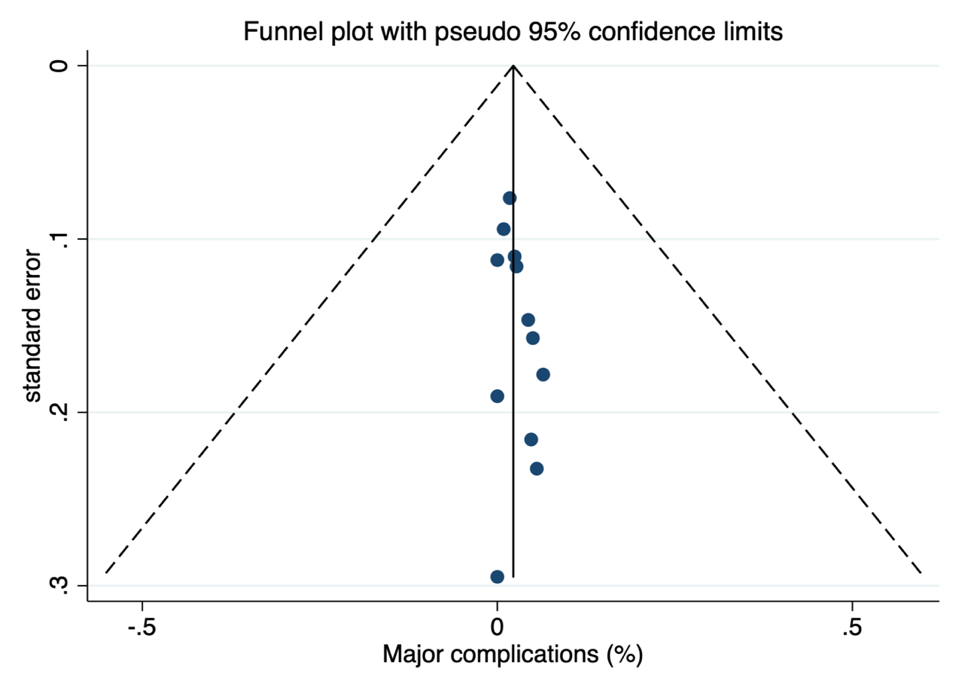


**REOPERATION RATES**

(Supplementary material. 7)

S7A. Data of Egger’s test


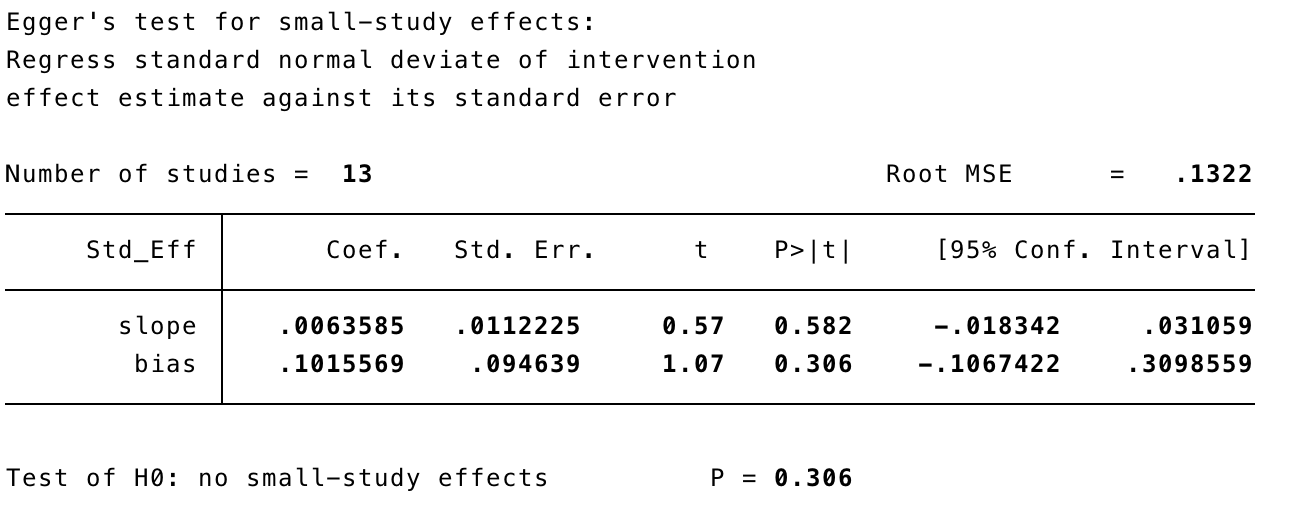


S7B. Funnel plot showing publication bias; reoperation % (X-axis) with it is standard error (Y-axis)


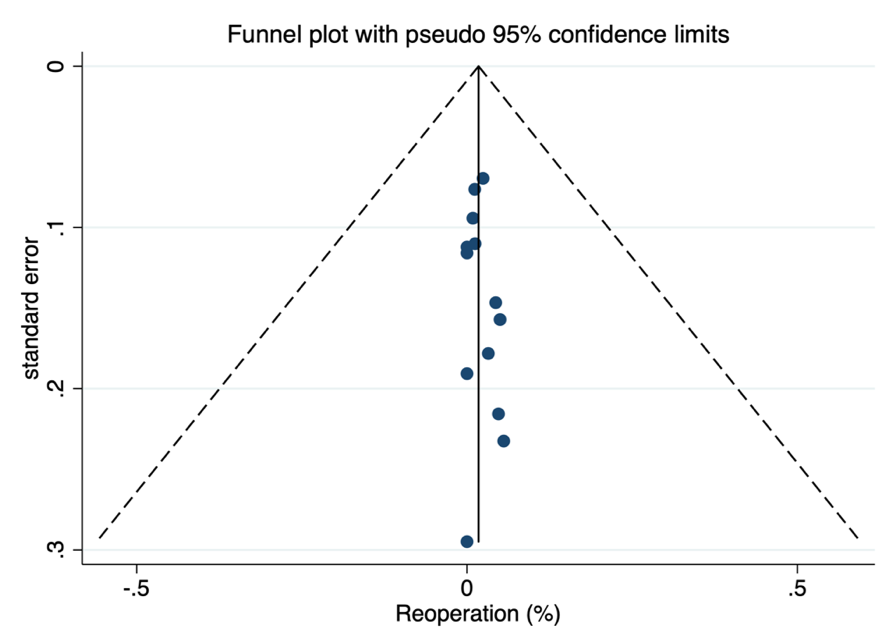


**READMISSION RATE**

(Supplementary material. 8)

**S**8A. Data of Egger’s test

**
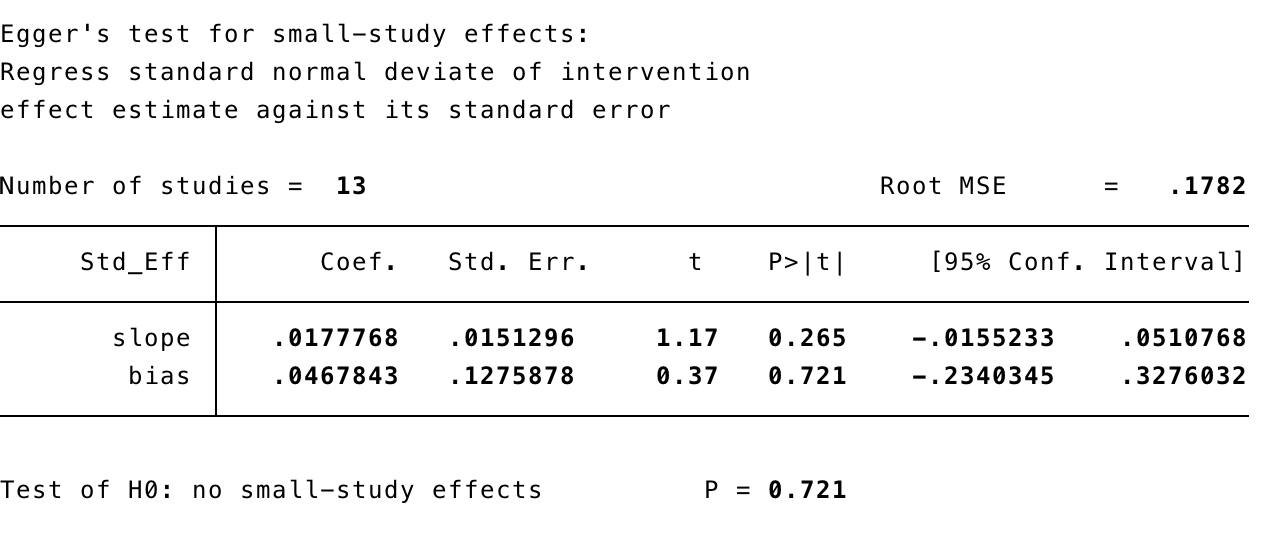
**

S8B. Funnel plot showing publication bias; readmission % (X-axis) with it is standard error (Y-axis)

**
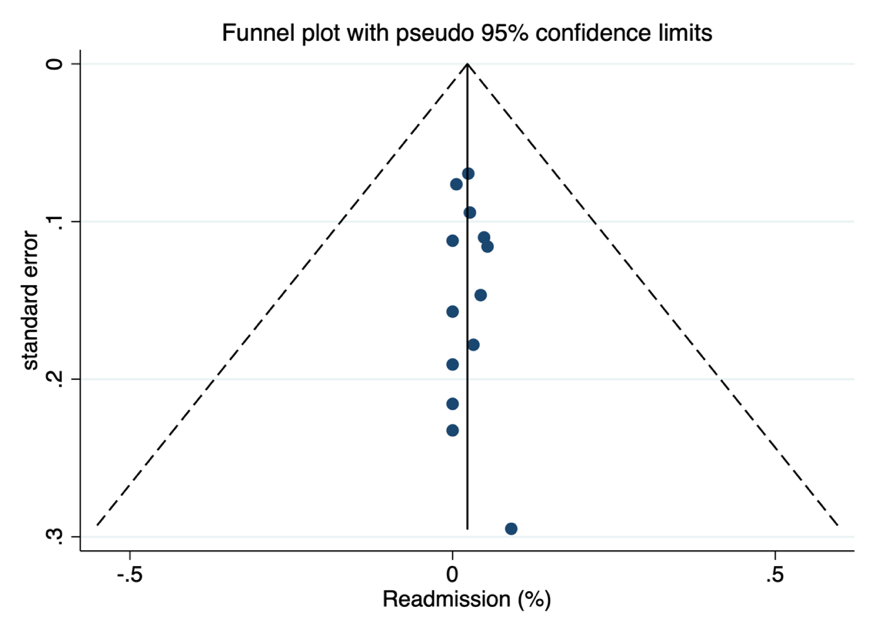
**

**RECURRENCE RATE**

(Supplementary material.9)

S9A. Data of Egger’s test


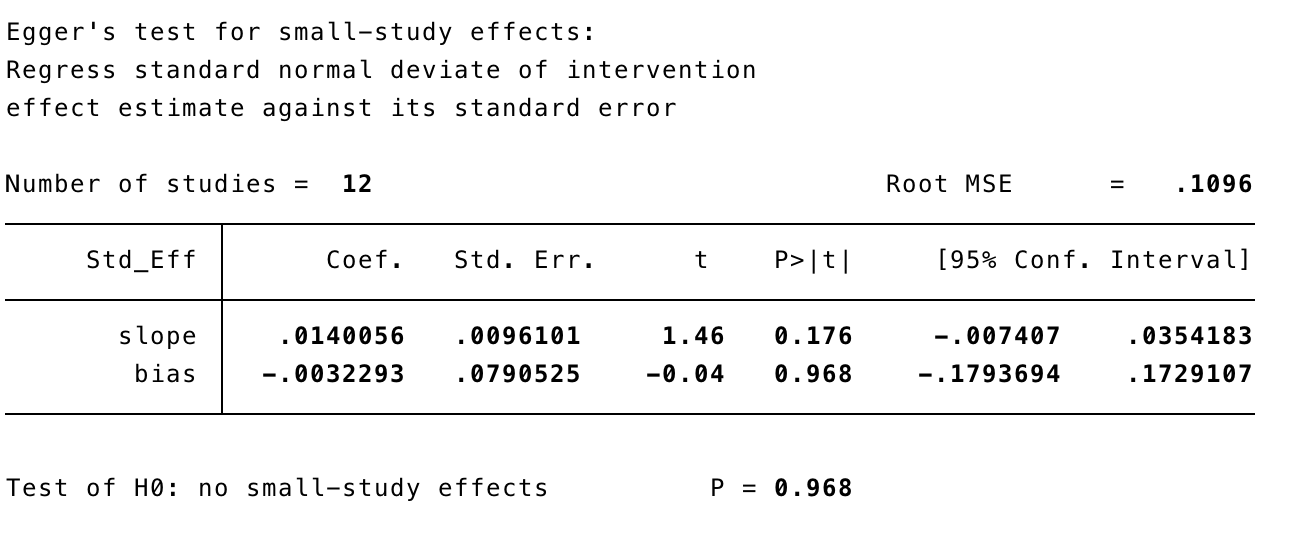


S9B. Funnel plot showing publication bias; recurrence % (X-axis) with it is standard error (Y-axis)


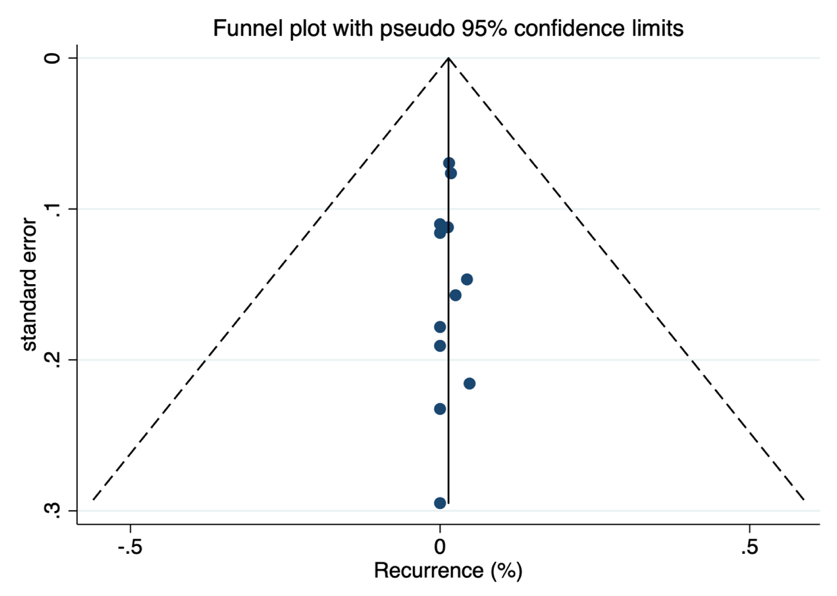


2. SUPPLEMENTARY TABLES

| **Table 1S.** Studies excluded from the meta-analysis | |
| --- | --- |
| **Study** | **Cause of exlusion** |
| Outcomes after Ventral Hernia Repair Using the Rives-Stoppa, Endoscopic, and Open Component Separation Techniques. | Duplicate or incomplete data |
| A preliminary multicenter evaluation of endoscopic sublay repair for ventral hernia from China. | Different or not defined technique |
| Open retromuscular versus laparoscopic ventral hernia repair for medium-sized defects: where is the value? | Different or not defined technique |
| What is the outcome of the open IPOM versus sublay technique in the treatment of larger incisional hernias?: A propensity score-matched comparison of 9091 patients from the Herniamed Registry. | Different or not defined technique |
| Incisional hernia prosthetic surgery: a prospective study comparing laparoscopic and open techniques. | Other lenguage |
| Retromuscular Approach in Ventral Hernia Repair - Endoscopic Rives-Stoppa Procedure. | Other lenguage |
| The current state of robotic retromuscular repairs-a qualitative review of the literature. | Duplicate or incomplete data |
| A comparison of robotic mesh repair techniques for primary uncomplicated midline ventral hernias and analysis of risk factors associated with postoperative complications. | Duplicate or incomplete data |
| Robotic intraperitoneal onlay versus totally extraperitoneal (TEP) retromuscular mesh ventral hernia repair: A propensity score matching analysis of short-term outcomes. | Duplicate or incomplete data |
| Short-term outcomes for open and laparoscopic midline incisional hernia repair: a randomized multicenter controlled trial: the ProLOVE (prospective randomized trial on open versus laparoscopic operation of ventral eventrations) trial. | Different or not defined technique |
| Laparoscopic incisional hernia repair: our experience and review of the literature. | Other lenguage |
| Comparative analysis of open and robotic transversus abdominis release for ventral hernia repair. | Different or not defined technique |
| Lateral approach totally extraperitoneal (TEP) robotic retromuscular ventral hernia repair. | Duplicate or incomplete data |
| Robotic retromuscular ventral hernia repair and transversus abdominis release: short-term outcomes and risk factors associated with perioperative complications. | Duplicate or incomplete data |
| The endoscopic retromuscular repair of ventral hernia: the eTEP technique and early results. | Inclusion of other type of hernia |
| Which should be the gold standard laparoscopic technique for handling Spigelian hernias? | Inclusion of other type of hernia |
| Optimized approach to the surgical treatment of patients with large and giant postoperative ventral hernia. | Other lenguage |
| Comparative analysis of perioperative outcomes of robotic versus open transversus abdominis release. | Different or not defined technique |
| Totally Endoscopic Sublay Anterior Repair for Ventral and Incisional Hernias. | Different or not defined technique |
| Extraperitoneal laparoscopic ventral hernia repair: one step beyond. | Inclusion of other type of hernia |
| Early operative outcomes of endoscopic (eTEP access) robotic-assisted retromuscular abdominal wall hernia repair. | Inclusion of other type of hernia |
| Modified robot assisted Rives/Stoppa videosurgery for midline ventral hernia repair. | Other lenguage |
| Reducing Length of Stay Using a Robotic-assisted Approach for Retromuscular Ventral Hernia Repair: A Comparative Analysis From the Americas Hernia Society Quality Collaborative. | Different or not defined technique |
| Rives-Stoppa incisional hernia repair combined with laparoscopic separation of abdominal wall components: a novel approach to complex abdominal wall closure. | Different or not defined technique |
| Robotic and hybrid robotic transversus abdominis release may be performed with low length of stay and wound morbidity. | Different or not defined technique |
| Hybrid versus open retromuscular abdominal wall repair: early outcomes. | Different or not defined technique |
| Retromuscular sutured incisional hernia repair: a randomized controlled trial to compare open and laparoscopic approach. | Different or not defined technique |
| Simultaneous laparoscopic totally extraperitoneal repair for concurrent ipsilateral spigelian and indirect inguinal hernia. | Inclusion of other type of hernia |
| Totally endoscopic sublay (TES) repair for midline ventral hernia: surgical technique and preliminary results. | Different or not defined technique |
| A novel technique of lumbar hernia repair using bone anchor fixation. | Inclusion of other type of hernia |
| Hybrid robotic transversus abdominis release versus open: propensity-matched analysis of 30-day outcomes. | Different or not defined technique |
| Endoscopic totally extraperitoneal approach (TEA) technique for primary ventral hernia repair. | Different or not defined technique |
| Incisional hernia surgery: report on 283 cases. | Different or not defined technique |
| Laparoscopic Stapled Sublay Repair with Self-Gripping Mesh: A Simplified Technique for Minimally Invasive Extraperitoneal Ventral Hernia Repair. | Different or not defined technique |

| Table 2S. Preoperative characteristics of patients undergoing minimally invasive eTEP approach for ventral hernia repair. | | | | | | | | |
| --- | --- | --- | --- | --- | --- | --- | --- | --- |
| Author | **Age** | **Sex ratio (M/F)**^b^ | **BMI (kg/m^2^)** | **Primary/Incisional** ^b^ | **Midline/Lateral** ^b^ | **ASA score** | **Size defect (width cm)** | **Mesh area (cm^2^)** |
| Kumar N et al. | 44.24 ± 7.45 | 63.0/37.0 | 28.60 + 4.15 | 60.9/59.9 | 100/0.0 | NR | 3.89 + 0.85 | NR |
| Ngo P et al. | 55.8 ± 11.5 | 67.9/32.1 | 27.4 ± 3.35 | NR | 100/0.0 | 1.44 ± 0.64 | NR | 225 ± 75 |
| Salido S et al. | 58 ± 14.5 | 52.5/47.5 | 30 ± 4.5 | 30/70 | 75/25 | 2.02 ± 0.47 | 4.05 ± 1.58 | 400 ± 199 |
| Prakhar G et al. | 49.34 ± 10.75 | 39.8/60.2 | 29.2 ± 4.1 | 56.1/43.9 | 100/0.0 | NR | NR | 397.56 ± 208.83 |
| Mitura K et al. | 51.4 ± 9 | 36.4/63.6 | 30.2 ± 2.25 | 36.4/63.6 | 90.9/9.1 | 1.81 ± 0.6 | 5.8 ± 1 | 18.3 ± 1.38 |
| Sanna A et al. | 57.05 ± 11.96 | 44.4/55.6 | 26.6 ± 1.93 | 33.3/67 | 100/0.0 | 2.23 ± 0.4 | NR | 267.47 ± 84.94 |
| Morrell ALG et al. | 51.1 ± 8.83 | 47.3/52.7 | 29.1 ± 4.45 | 55.4/44.6 | NR | 1.83 ± 0.8 | 5.6 ± 3.83 | 456.5 ± 80 |
| Kudsi OY et al. | 57 ± 14.5 | 51.2/48.8 | 31.8 ± 7.2 | 39.0/61.0 | NR | 2 ± 0.17 | 18.8 ± 10.5 | 300 ± 62.5 |
| Köhler G et al. | 61.6 ± 15.35 | 45.2/54.8 | 28.6 ± 4.08 | 71.0/29.0 | 100/0.0 | 2.1 ± 0.9 | 3.6 ± 1.6 | 420 ± 114 |
| Baig SJ et al. | 54.67 ± 13.05 | 28.6/71.4 | 28.57 ± 4.14 | NR | 100/0.0 | NR | 6.65 ± 1.95 | 535 ± 193.35 |
| Penchev D et al. | 58.7 ± 11.7 | 55.6/44.4 | 25.1 ± 3.9 | 29.6/70.4 | 100/0.0 | NR | NR | 428.4 ± 220.6 |
| Lu R et al. | 52 ± 13.73 | 55.8/44.2 | 32.85 ± 6.78 | NR | NR | 2.25 ± 0.52 | 6.3 ± 2.24 | 516.9 ± 243.67 |
| Belyansky I et al. | 54.9 ± 13.9 | 43.0/57.0 | 31.1 ± 6.0 | NR | NR | 2.3 ± 0.6 | NR | 634.4 ± 319.7 |
| *Data are expressed as mean (standard deviation or range) unless otherwise specified.*  *Abbreviations: eTEP,* enhanced view totally extraperitoneal; *BMI,* body mass index; *ASA,* American Society of anaesthesiologist; *NR* data not reported  ^b^ no. (%) | | | | | | | | |

| Table 3S. Perioperative characteristics of patients undergoing minimally invasive eTEP approach for ventral hernia repair. | | | | | | | | |
| --- | --- | --- | --- | --- | --- | --- | --- | --- |
| Author | **No. patients** | **Operative time (mins)^a^** | **Surgical approach** | **Surgical technique** | **Blood loss (ml)** | **LOS (days)** | **I.O complications** | **Conversion** |
| Kumar N et al. | 46 | 107.52 +23.44 | Laparoscopic | 46 RS | <50 | 1.11+ 0.31 | 0 (0.0) | 0 (0.0) |
| Ngo P et al. | 112 | 75 ± 40 | Laparoscopic | 112 RS | NR | NR | 5 (4.5) | 5 (4.5) |
| Salido S et al. | 40 | 126 ± 36 | Laparoscopic | 24 RS + 16 TAR | NR | 1 ± 4.25 | 4 (10.0) | 2 (5.0) |
| Prakhar G et al. | 171 | 176.75 ± 62.42 | Laparoscopic | 121 RS + 50 TAR | 78.7 ± 24.4 | 2.18 ± 1.27 | 6 (3.5) | 4 (2.3) |
| Mitura K et al. | 11 | 204 ± 34.25 | Laparoscopic | 11 RS | NR | 3.4 ± 1 | 1 (9.1) | 0 (0.0) |
| Sanna A et al. | 18 | 125.64 ± 27.21 | Laparoscopic | 14 RS + 4 TAR | NR | 2.76 ± 0.75 | NR | NR |
| Morrell ALG et al. | 74 | 174.4 ± 39.2 | Robotic | 66 RS + 8 TAR | NR | 1.5 ± 0.66 | 0 (0.0) | 0 (0.0) |
| Kudsi OY et al. | 82 | 106.5 ± 17.8 | Robotic | 36 RS + 46 TAR | 5 (5–20) | 0 ± 0.83 | 0 (0.0) | 0 (0.0) |
| Köhler G et al. | 31 | 128 ± 35 | Laparoscopic | 31 RS | NR | 3 ± 1.5 | 1 (3.2) | 1 (3.2) |
| Baig SJ et al. | 21 | 176.48 ± 40.95 | Laparoscopic | 9 RS + 12 TAR | NR | 2.67 ± 0.75 | NR | NR |
| Penchev D et al. | 27 | 186 ± 62 | Laparoscopic | 27 RS | <100* | 2.9 (NR) | 1 (3.7) | 0 (0.0) |
| Lu R et al. | 206 | 147.55 ± 40.26 | Laparoscopic  + Robotic | NR | NR | 0.15 ± 0.73 | NR | NR |
| Belyansky I et al. | 79 | 218.9 ± 111.2 | Laparoscopic | 38 RS + 41 TAR | 52.6 ± 39.5 | 1.8 ± 1.8 | 2 (2.5) | 2 (2.5) |
| *Data are expressed as n (%) unless otherwise specified.*  *Abbreviations: eTEP,* enhanced view totally extraperitoneal; *RS,* Rives Stoppa; *TAR,* transversus abdominis release; *LOS,* length of stay; *I.O,* intraoperative; *NR,* data not reported  ^a^ *Mean (standard deviation)*  **except 1 patient* | | | | | | | | |

| Table 4S. Short term-postoperative outcomes of patients undergoing minimally invasive eTEP approach for ventral hernia repair. | | | | | | | | | |
| --- | --- | --- | --- | --- | --- | --- | --- | --- | --- |
| Author | **No. patients** | **Surgical site infection (SSI)** | **Seroma** | **Hematoma** | **Readmission** | **Major complication**  **(DC III/IV)** | **Reoperation** | **Follow up (month)^a^** | **Recurrence** |
| Kumar N et al. | 46 | 0 (0.0) | 6 (13.0) | 0 (0.0) | 2 (4.3) | 2 (4.3) | 2 (4.3) | 6 | 2 (4.3) |
| Ngo P et al. | 112 | 0 (0.0) | 1 (0.9) | 1 (0.9) | 3 (2.7) | 1 (0.9) | 1 (0.9) | NR | NR |
| Salido S et al. | 40 | NR | 1 (2.5) | 2 (5.0) | 0 (0.0) | 2 (5.0) | 2 (5.0) | 10 ± 3.75 | 1 (2.5) |
| Prakhar G et al. | 171 | 5 (2.9) | 4 (2.3) | 1 (0.6) | 1 (0.6) | 3 (1.8) | 2 (1.2) | 6 | 3 (1.8) |
| Mitura K et al. | 11 | 0 (0.0) | 2 (18.2) | 1 (9.1) | 1 (9.1) | 0 (0.0) | 0 (0.0) | 7.2 ± 4 | 0 (0.0) |
| Sanna A et al. | 18 | 0 (0.0) | 2 (11.1) | 1 (5.6) | 0 (0.0) | 1 (5.6) | 1 (5.6) | 1 | 0 (0.0) |
| Morrell ALG et al. | 74 | 1 (1.3) | 10 (13.5) | 4 (5.4) | 4 (5.4) | 2 (2.7) | 0 (0.0) | 7.72 ± 2.57 | 0 (0.0) |
| Kudsi OY et al. | 82 | 1 (1.2) | 3 (3.7) | 1 (1.2) | 4 (4.9) | 2 (2.4) | 1 (1.2) | 24.6 ± 12.7 | 0 (0.0) |
| Köhler G et al. | 31 | 1 (3.2) | NR | NR | 1 (3.2) | 2 (6.4) | 1 (3.2) | 8 | 0 (0.0) |
| Baig SJ et al. | 21 | 0 (0.0) | 1 (4.8) | 0 (0.0) | 0 (0.0) | 1 (4.8) | 1 (4.8) | 2 | 1 (4.8) |
| Penchev D et al. | 27 | 0 (0.0) | 4 (14.8) | 0 (0.0) | 0 (0.0) | 0 (0.0) | 0 (0.0) | 1 | 0 (0.0) |
| Lu R et al. | 206 | NR | 8 (3.9) | 3 (1.5) | 5 (2.4) | NR | 5 (2.4) | 5.6 ± 5.42 | 3 (1.5) |
| Belyansky I et al. | 79 | 0 (0.0) | 2 (2.5) | 0 (0.0) | 0 (0.0) | 0 (0.0) | 0 (0.0) | 10.7 ± 4.1 | 1 (1.3) |
| *Data are expressed as n (%) unless otherwise specified.*  *Abbreviations: eTEP,* enhanced view totally extraperitoneal; *RS,* Rives Stoppa; *TAR,* transversus abdominis release; *LOS,* length of stay; *I.O,* intraoperative; *NR,* data not reported  ^a^ *Mean (standard deviation)* | | | | | | | | | |

| Table 5S. Methodological items for non-randomized studies (MINORS) score for studies included | | | | | | | | | | | | | |
| --- | --- | --- | --- | --- | --- | --- | --- | --- | --- | --- | --- | --- | --- |
| Author | **A clearly stated aim** | **Inclusion of consecutive patients** | **Prospective collection of data** | **Endpoints appropriate to the aim of the study** | **Unbiased assessment of the study endpoint** | **Follow-up period appropriate to the aim of the study** | **Loss to follow up less than 5%** | **Prospective calculation of the study size** | **An adequate control group** | **Contemporary groups** | **Baseline equivalence of groups** | **Adequate statistical analyses** | **Total score** |
| Kumar N et al. | 2 | 2 | 2 | 2 | 0 | 1 | 2 | 0 | 2 | 2 | 2 | 2 | **19** |
| Ngo P et al. | 2 | 2 | 2 | 2 | 0 | 0 | 0 | 0 | NA | NA | NA | NA | **8** |
| Salido S et al. | 2 | 2 | 2 | 2 | 0 | 2 | 1 | 0 | NA | NA | NA | NA | **11** |
| Prakhar G et al. | 2 | 2 | 0 | 1 | 0 | 1 | 1 | 0 | NA | NA | NA | NA | **7** |
| Mitura K et al. | 2 | 2 | 2 | 2 | 0 | 1 | 0 | 0 | NA | NA | NA | NA | **9** |
| Sanna A et al. | 2 | 2 | 0 | 2 | 0 | 1 | 2 | 0 | NA | NA | NA | NA | **9** |
| Morrell ALG et al. | 2 | 2 | 2 | 2 | 0 | 2 | 1 | 0 | NA | NA | NA | NA | **11** |
| Kudsi OY et al. | 2 | 2 | 2 | 1 | 0 | 2 | 0 | 0 | 2 | 2 | 2 | 2 | **17** |
| Köhler G et al. | 2 | 2 | 2 | 2 | 0 | 2 | 1 | 0 | NA | NA | NA | NA | **11** |
| Baig SJ et al. | 2 | 2 | 0 | 2 | 0 | 1 | 0 | 0 | NA | NA | NA | NA | **7** |
| Penchev D et al. | 2 | 2 | 0 | 2 | 0 | 1 | 2 | 0 | 2 | 2 | 2 | 2 | **17** |
| Lu R et al. | 2 | 2 | 2 | 2 | 0 | 2 | 1 | 0 | 2 | 2 | 1 | 2 | **18** |
| Belyansky I et al. | 2 | 2 | 2 | 2 | 0 | 2 | 0 | 0 | NA | NA | NA | NA | **10** |
| *Abbreviations:* NA no aplicable | | | | | | | | | | | | | |


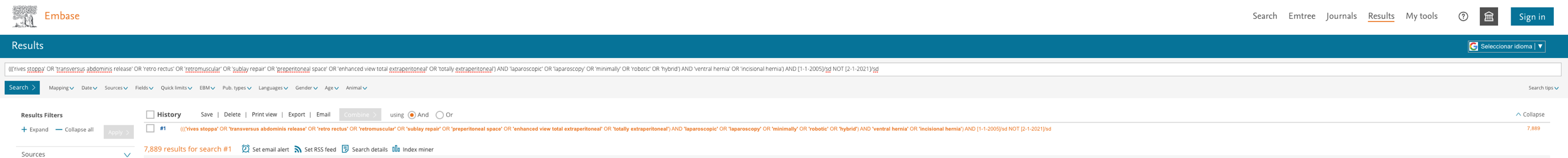
3. SEARCH STRATEGY


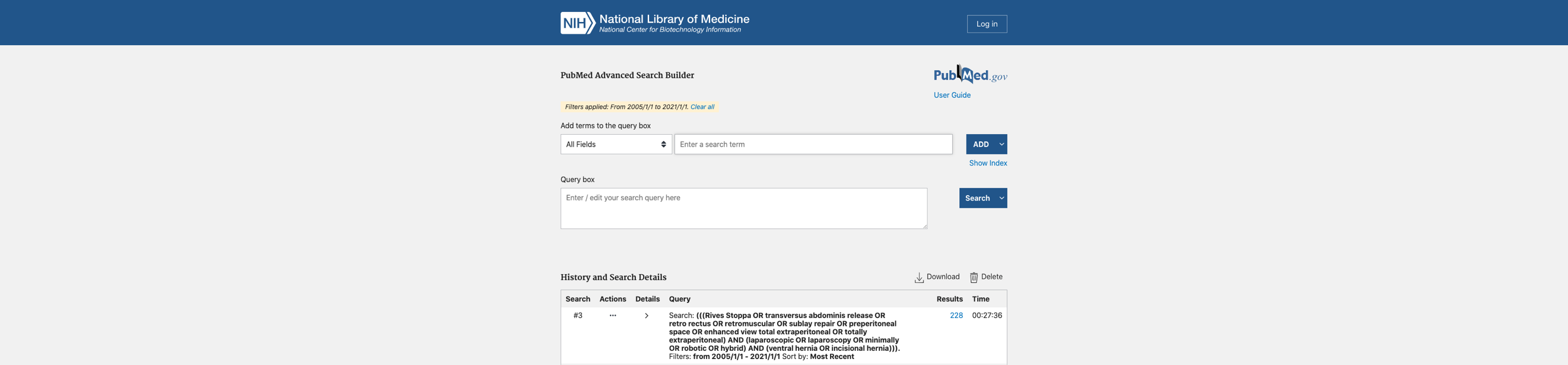


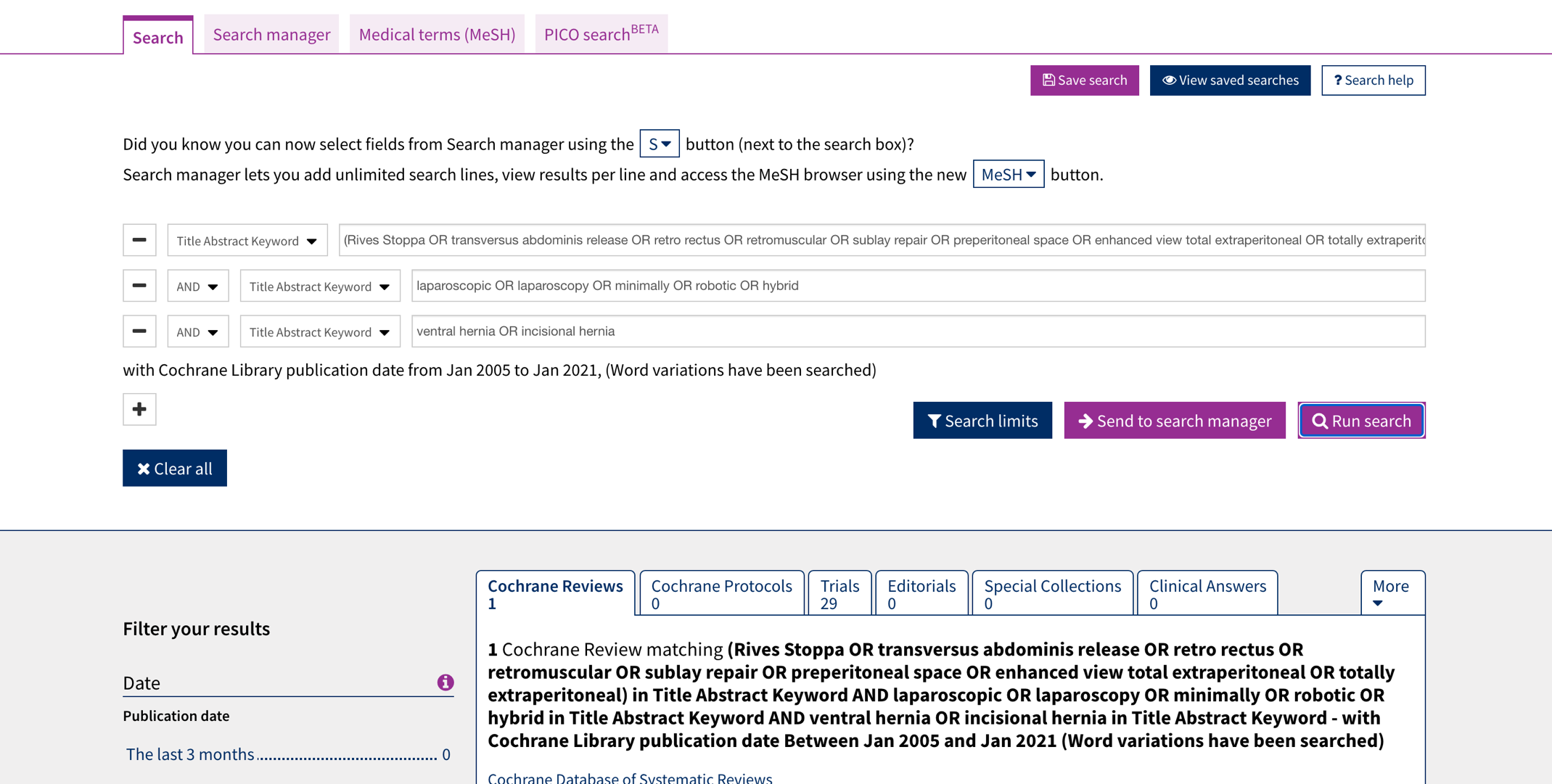

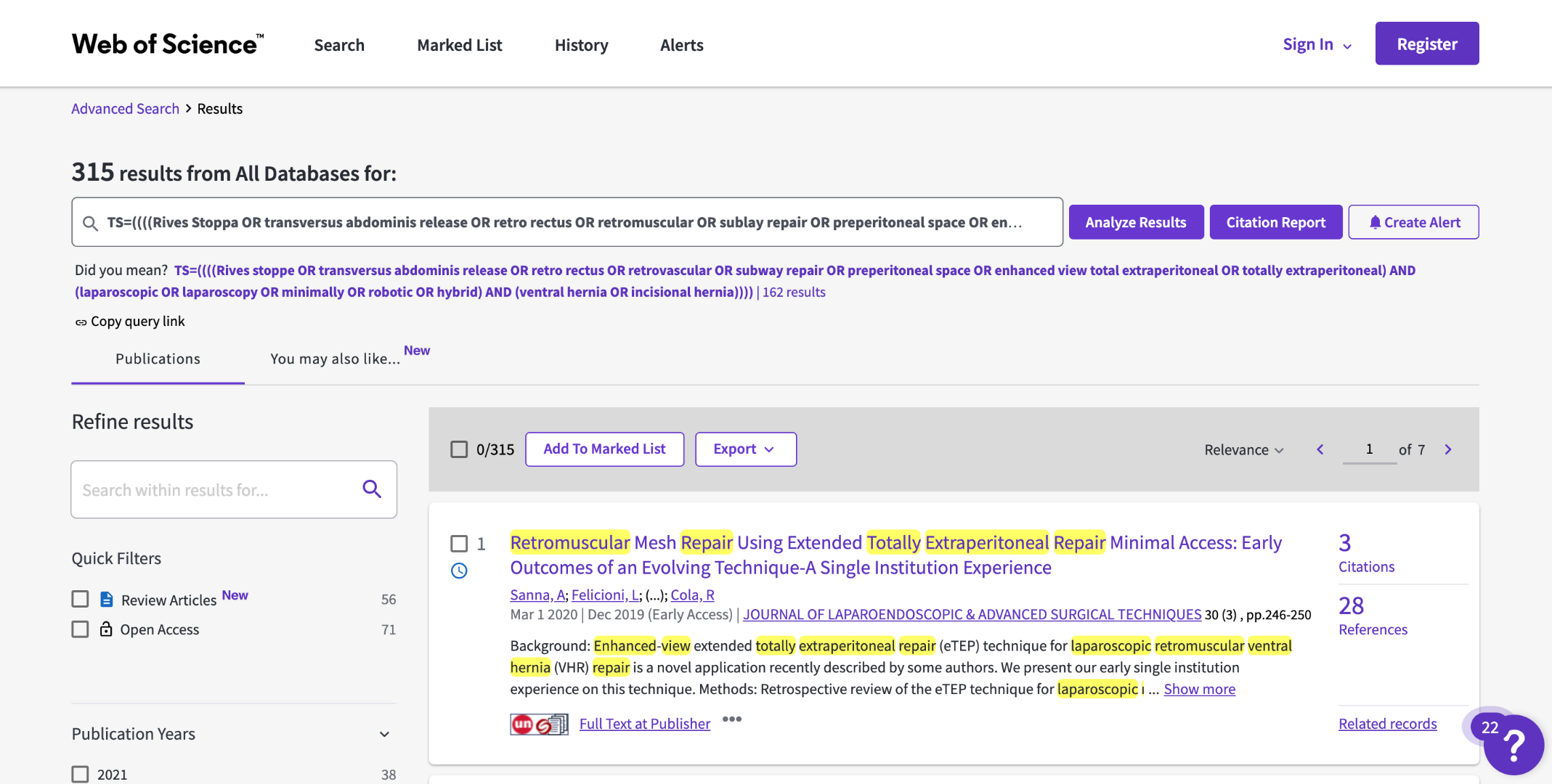

Supplement: Supplementary file 1 — Supplementary file1 (DOCX 2377 KB) [file 10029_2021_2557_MOESM1_ESM.docx]
